# Joint Toxicity and Interaction of Carbon-Based Nanomaterials with Co-Existing Pollutants in Aquatic Environments: A Review

**DOI:** 10.3390/ijms252111798

**Published:** 2024-11-02

**Authors:** Konstantin Pikula, Seyed Ali Johari, Ralph Santos-Oliveira, Kirill Golokhvast

**Affiliations:** 1 Polytechnical Institute, Far Eastern Federal University, 10 Ajax Bay, Russky Island, 690922 Vladivostok, Russia; pikula_ks@dvfu.ru; 2 Department of Fisheries, Faculty of Natural Resources, University of Kurdistan, Pasdaran St, Sanandaj 66177-15175, Kurdistan, Iran; 3 Laboratory of Synthesis of Novel Radiopharmaceuticals and Nanoradiopharmacy, Nuclear Engineering Institute, Brazilian Nuclear Energy Commission, Rio de Janeiro 21941-906, Brazil; 4 Laboratory of Nanoradiopharmaceuticals and Radiopharmacy, State University of Rio de Janeiro, Rio de Janeiro 23070-200, Brazil; 5 Siberian Federal Scientific Center of Agrobiotechnology RAS, 2b Centralnaya, Presidium, 633501 Krasnoobsk, Russia; 6 Advanced Engineering School “Agrobiotek”, Tomsk State University, 36 Lenina Avenue, 634050 Tomsk, Russia

**Keywords:** carbon nanotubes, ecological risk assessment, ecotoxicology, graphene, mixture toxicity modeling, nanotoxicology, quantitative structure–activity relationship, synergistic effects

## Abstract

This review paper focuses on the joint toxicity and interaction of carbon-based nanomaterials (CNMs) with co-existing pollutants in aquatic environments. It explores the potential harmful effects of chemical mixtures with CNMs on aquatic organisms, emphasizing the importance of scientific modeling to predict mixed toxic effects. The study involved a systematic literature review to gather information on the joint toxicity and interaction between CNMs and various co-contaminants in aquatic settings. A total of 53 publications were chosen and analyzed, categorizing the studies based on the tested CNMs, types of co-contaminants, and the used species. Common test models included fish and microalgae, with zebrafish being the most studied species. The review underscores the necessity of conducting mixture toxicity testing to assess whether the combined effects of CNMs and co-existing pollutants are additive, synergistic, or antagonistic. The development of in silico models based on the solid foundation of research data represents the best opportunity for joint toxicity prediction, eliminating the need for a great quantity of experimental studies.

## 1. Introduction

Considering the great opportunity for the application of carbon-based nanomaterials (CNMs), it is not surprising that the global market of CNMs has been rapidly growing for the last two decades. A recent report (August 2023) by the analytical company Precedence Research Pvt. Ltd. (Canada and India) estimated the global CNMs market size at USD 3.6 billion in 2022 and predicted it to rise to USD 40.71 billion by 2032, with a notable compound annual growth rate (CAGR) of 27.5% during the forecast period 2023 to 2032 (https://www.precedenceresearch.com/carbon-nanomaterials-market, accessed on 30 August 2024). The forecast assumed that there would be the most remarkable CAGR of 16.6% for carbon nanotubes (CNTs) during the analyzed period, with the highest growth of CNMs application in the medical and healthcare segment (17.2% CAGR). From a safety point of view, it should be highlighted that the impressive extension of CNM production and application will inevitably lead to an increase in the frequency and volume of contact between these materials and humans or the environment [1,2,3].

Aquatic ecosystems could be considered the most vulnerable environmental entity. The aquatic environment experiences the impact of natural CNMs released by dust storms, forest fires, volcanic activities, and different anthropogenic combustion processes [4]. Engineered CNMs can appear in water intentionally, since they are used in groundwater remediation, or unintentionally, as a result of biomedical or other types of applications [5,6,7]. It is important to note that the release of engineered CNMs into the environment could occur at each stage of the life cycle, such as its production, storage, application, and disposal [8]. Moreover, the atmospheric release of nanomaterials, and their infiltration and accumulation in the soil means that they can reach a water body via sedimentation and surface wash [9,10].

The environmental risk assessment for nanomaterials, including CNMs, has been studied for more than two decades [11]. However, understanding the toxicity and their effective risk management requires considering many properties of the materials, such as the multiple variations and possible combinations of their properties, environmental conditions, and the different sensitivities of exposed species and organisms. Among these parameters, the interaction of CNMs with other chemicals and compounds in aquatic media represents one of the less studied issues.

The specific objective of this study was to systematically review the existing data on the interaction and joint toxicity of CNMs with other emerging aquatic contaminants, aiming to provide a background and present the state of the art in this topic. The following sections of this paper will present (1) an overview of CNMs’ history, application, and risk assessment challenges, (2) a discussion on predictive models for mixture toxicity assessment and their applicability to CNMs, (3) a collection and systematic review of currently available research data of CNM interaction and their joint toxicity with common aquatic pollutants, such as heavy metals, pesticides, organic contaminants, and others.

## 2. Carbon-Based Nanomaterials: History, Risks, and Challenges

### 2.1. History, Classification, and Application of Carbon-Based Nanomaterials

Natural carbon-based nanomaterials (CNMs) have been a part of the environment for billions of years. The sources of natural CNMs include incomplete combustion in forest fires and volcanic activity, earthquakes, wind erosion, and others [12]. Early human activities additionally brought incidental CNMs into the environment through the combustion by-products of smoke and campfires, and the volume of these emissions has been rapidly increasing since the beginning of the Industrial Revolution [13]. The next milestone was the rise of nanotechnology with the expansion of engineered nanomaterials, which possess unique and tunable properties and provide great opportunities, meaning that they are involved in many areas of industry and daily life [14]. A comprehensive overview of the prehistorical, ancient, medieval, and modern formation, production, and application of natural, incidental, and engineered nanomaterials, including their classification, properties, and associated risks, is provided in the work of Barhoum et al. (2022) [15].

**Figure 1:**
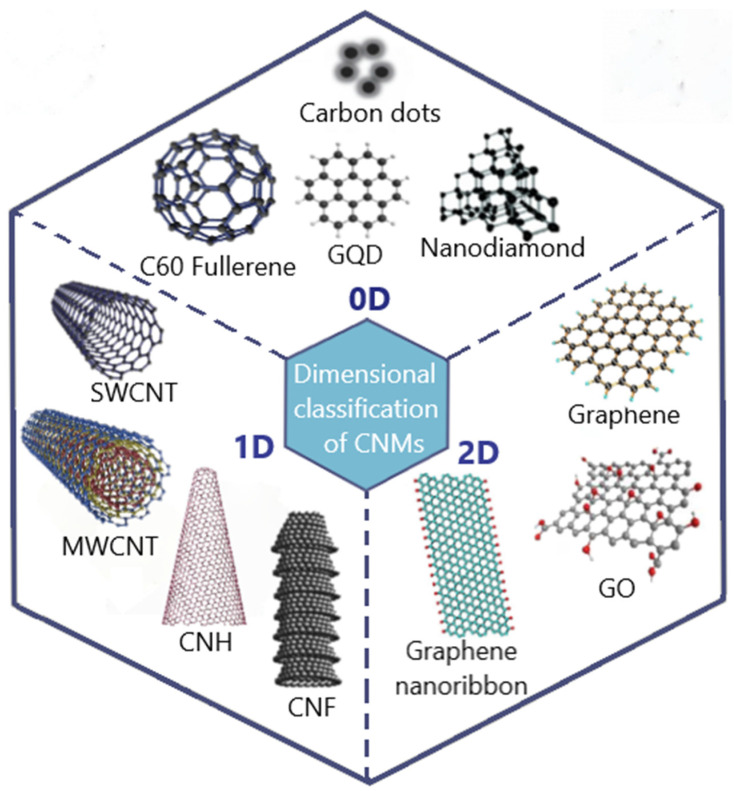
Dimensional classification of CNMs. GQD, graphene quantum dot; SWCNT, single-walled carbon nanotubes; MWCNT, multi-walled carbon nanotubes; CNH, carbon nanohorn; CNF, carbon nanofiber; GO, graphene oxide.

Conventionally, graphite and diamond represent the most common and studied carbon allotropes. The discovery of fullerenes in 1985 by Kroto et al. [16], the clarification of the carbon nanotube (CNT) structure by Sumio Iijima in 1991 [17], and the discovery of graphene by Geim and Novoselov in 2004 [18] and their exploration of the properties of this material in the series of their works from around 2010 [19,20,21] have garnered great excitement in the scientific community. Currently, in addition to graphene, CNTs, and fullerenes, the family of CNMs includes a variety of products with remarkable properties, such as carbon dots (CDs), nanodiamonds (NDs), carbon nanohorns (CNHs), carbon nanofibers (CNFs), etc. [22]. The properties of these novel materials depend on hybridization and bonding between C atoms, crystallinity, size, shape, structure, surface properties, and others. According to dimensional classification, the different types of CNMs are divided into zero-dimensional (0D), one-dimensional (1D), or two-dimensional (2D) materials, where the number reflects the number of dimensions above 100 nm [23]. Several examples of the dimensional distribution of CNMs are represented in Figure 1.

The group of 0D materials, such as fullerenes, CDs, graphene quantum dots, and NDs have high solubility in water, contrary to the other types of CNMs which predominantly form unstable dispersions [22]. These materials have remarkable optic properties, such as photoluminescence, which can be further tuned with surface modification [24].

The group of 1D materials is represented by CNTs, CNFs, and CNHs. Carbon nanotubes demonstrate unique electrical properties useful for advanced electronics, electrical transport, energy storage, biomolecular sensing, and many other aspects [25]. Moreover, a great surface area and porous structure make CNTs a promising substrate for targeted drug delivery, photodynamic and contrast therapy, and surface functionalization, further extending their possible applications [26,27]. Carbon nanohorns (also known as nanocones), when compared to CNTs, do not have potentially toxic metal catalysts in their synthesis and can be produced at room temperature; however, they have low symmetry and tend to aggregate into spherical clusters [28]. Carbon nanohorns have been suggested to have more effective surface functionalization than CNTs and have a great variety of applications [28].

The group of 2D materials includes graphene and its derivatives, namely graphene oxide (GO), reduced graphene oxide (rGO), graphene nanoribbons, and graphitic multilayered nanosheets [22]. The rapid increase in graphene research has become possible due to the relatively simple and cheap laboratory production of high-quality graphene with exceptional characteristics, including mechanical stiffness, strength and elasticity, and very high electrical and thermal conductivity, greater than any other materials [29]. Alongside the other CNMs, graphene can successfully undergo surface modification. Due to the presence of oxygen groups, GO can interact with various molecules and can be applied for the removal of toxic gasses and the absorption of metal ions in water purification. Graphene oxide also finds biomedical applications in drug delivery and biosensing [30].

### 2.2. Environmental Safety and Risk Assessment of Carbon-Based Nanomaterials

Since the early 2000s, the rise of nanotechnology as an industry has been a cause of concern regarding the safety of nanoparticles [31,32]. In 2004, Donaldson et al. introduced a new subcategory of toxicology named ‘nanotoxicology’ [33], and then the family of Günter, Eva, and Jan Oberdörster played an important role in defining and formulating the main principles of this discipline [34,35]. It should be highlighted that the properties and safety of nanoparticles and bulk materials of the same chemical composition significantly differ, and even very subtle changes in size, morphology, surface properties, or other characteristics of nanosized particles can dramatically change their toxicological profile [36]. Thus, nanoparticle risk assessment appears to be a very challenging task, especially considering the variety of different types and modifications of nanomaterials.

Several studies have made efforts to model and compute the risks of the combination of existing aquatic contamination with CNMs. Hong et al. (2022) calculated the predicted environmental concentration of graphene and graphene-family nanomaterials for surface water in the EU, and it was estimated that the obtained value would reach 1.4 ng/L by 2030 [37]. The used model stated that the concentration of graphene and graphene-family nanomaterials will increase by more than 1000-fold between 2010 and 2030. Sun et al. (2014) obtained the predicted environmental concentration of CNTs and fullerenes in the surface water of the EU, which was 4.0 and 0.11 ng/L, respectively [38]. The growth of the CNMs market will lead to an increase in the risks for aquatic ecosystems. Moreover, it should be highlighted that the used calculations considered only background pollution, which did not include occasional releases.

For two decades, the classic approach of ecotoxicology was used to assess the effects of CNMs on representative species of aquatic biota [2,39]. Commonly used test species include bacteria, microalgae, crustacean, bivalves, other aquatic invertebrates, and fish. The ecotoxicological effect of different CNMs in aquatic species has been overviewed in several works [2,8,39,40].

The main benefits of this body of research work include a definition of toxicity thresholds and possible modes of toxic action (MoA) for different types of CNMs in different aquatic species. After the testing of nanomaterials in multiple single-species tests, these data can be consolidated to generate species sensitivity distributions (SSDs), which model the range of sensitivities of different species [41] and allow the further calculation of predicted no-effect concentrations (PNECs) for the whole biota [8]. For example, the PNEC values were reported for CNTs and fullerenes as 55.6 μg/L and 3.84 μg/L, respectively [42].

Despite the calculated PNECs considerably surpassing the predicted environmental concentration for the assessed materials (which is about several ng/L, as described above), there are possible risks associated with a variety of different nanomaterials, a lack in the knowledge of how the properties and characteristics of nanoparticles impact their toxicity, and the possible enhancement of toxicity caused by interaction with other existing background contaminants.

The conventional approach of ecotoxicology often does not take real-life circumstances into account, such as the transformation and aging of nanomaterials in aquatic media, the impact of background pollution, and the combined toxic action of nanomaterials with common aquatic pollutants. The listed aspects make risk assessment in aquatic nanotoxicology even more challenging and require careful examination.

Our previous work overviewed the main principles of the physical, chemical, and biological transformation of CNMs in aquatic environments, including biodegradation and bioaccumulation [3]. Apart from the physical and chemical characteristics of nanoparticles, further aspects that play a vital role in nanomaterials’ behavior include the pH and ionic strength of water, the presence and composition of natural organic matter (NOM), and absorption of proteins and the formation of so-called “protein corona”. It is very important to take these processes into account for accurate risk assessment.

The other challenging problem in environmental risk assessment is evaluating the joint toxic action of multiple co-existing pollutants. The understanding of interaction and mixture toxicity of chemicals requires a strong theoretical base for the developments of mixture toxicity predictive models and the collection of big amounts of experimental data to improve, train, and verify these predictive models.

The principles of mixture toxicity prediction, the applicability of the existing models to CNMs, and the available experimental data of CNMs joint toxicity with the other aquatic contaminants will be overviewed in the following sections.

## 3. Predictive Models for Mixture Toxicity Assessment

### 3.1. The Main Principles of Mixture Toxicity Modeling

In ecotoxicology and pharmacology, the joint toxic effects of chemical mixtures can be described using the terms additivity, synergism, and antagonism. In their work, Rodea-Palomares and co-authors (2015) summarized existing definitions and described these concepts [43]. In general, additivity is the idea that the combined effect of different chemicals is equal to the sum of the effects of each individual component. Synergism is the situation where the joint effect is greater than that estimated for additivity, while antagonism is the situation in which combined toxic response is less than estimated for additivity. Hence, the additivity provides a basis for the assessment of synergism and antagonism. Here, it should be highlighted that synergistic interactions represent the most harmful scenario, and even the chemicals considered as ‘safe’ could represent a serious threat to living organisms in this combination. From this point of view, the interaction between individual pollutants requires scientific-based modeling to predict the mixed toxic effect.

Based on the MoA of chemicals present in a mixture, the concept of additivity was expressed in two widely accepted mathematical models, namely Loewe’s model (similar MoA), also known as Concentration Addition (CA) [44], and Bliss’s model (different MoA), known as Independent Action (IA) [45].

Loewe’s CA model (Equation (1)) anticipates that compounds with similar MoA behave as higher doses of a single compound or as simple dilutions of one another [46]. Subsequently, if the mixture effect is well predicted by the CA model, the MoA of the chemicals can be considered similar [47]. The CA concept for “*n*” components is expressed in the following equation:

(1) $$\sum_{i=1}^{n}\frac{C_{i}}{D_{x,i}}=1$$

where C*_i_* is the concentration of chemical “*i*” in the mixture and *D_x_*_,*i*_ is the known concentration of “*i*” that causes “*x*” effect in individual exposure assay (LC50, EC50, EC10, NOEC and other endpoints can be used as “*x*”).

Bliss’s IA model (Equation (2)) assumes that the effects of chemicals with dissimilar MOE occur independently of each other and the overall effect can be predicted using the joint probability of occurrence for these effects [43,47,48]. The joint concentration of “*n*” components that cause the “*x*” effect can be predicted by the IA model as follows:

(2) $$D_{x,mix}=1-\prod_{i=1}^{n}(1-D_{x,i})$$

where ${(1-D}_{x,i})$ is the probability that “*x*” effect occurs through exposure to chemical “*i*”.

The important difference between these two models is that the total effect in the CA model will be affected, even if the individual components are below their no-effect concentration (NOEC) as the total dose will be changed; however, it would have no impact on the IA model, because in this case the effect is null. Bliss’s IA model is more rarely used in ecotoxicological studies because it cannot be used without the quantification data of the concentration–response relationships, and in real-life conditions, the independent action of chemicals is unrealistic [47].

**Figure 2:**
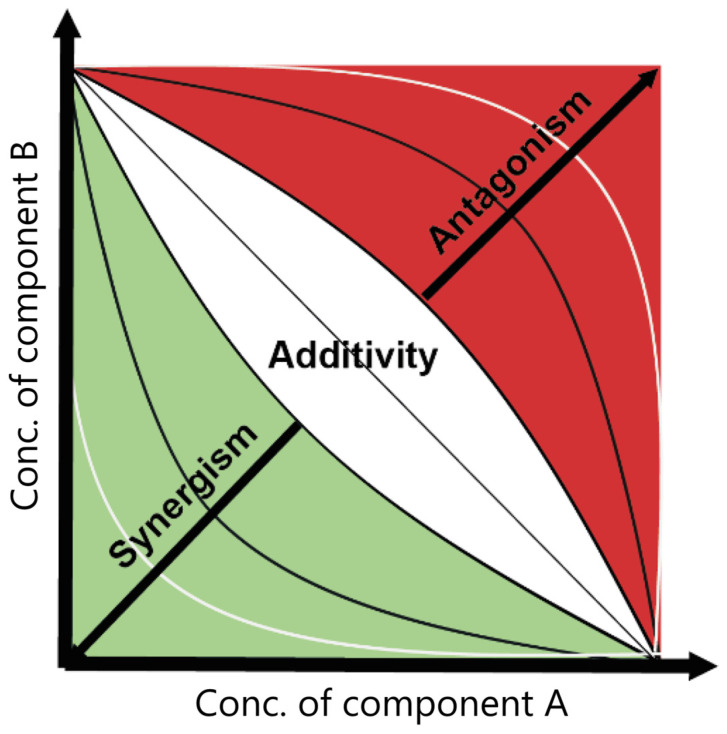
Schematic isobologram of the interaction between two chemicals. Image adapted from Cokol et al. [49]. Copyright 2011, EMBO and Macmillan Publishers Limited.

The application of the described models allows us to predict a joined effect of the mixture based on the known effects of individual components [47]. However, it is necessary to conduct mixture toxicity testing to evaluate whether the effect is additive or not. In the case where the observed result satisfies the used predictive model, the mixture effect is additive. Therefore, the interaction could be considered synergistic if the observed effect is significantly higher than expected, and it would be antagonistic when the observed effect is significantly lower than expected. A schematic visualization of interaction between two chemicals can be represented as inhibition in an isobologram based on the Loewe’s additivity model (Figure 2), where values near 0 indicate linearity (additivity), negative values indicate convexity (synergy), and positive values indicate concavity (antagonism) [49].

The statistical testing method for determining whether the observed mixture effect significantly deviates from the one predicted by additive models was described in the book of Masashi Kamo “Theories in Ecological Risk Assessment” (Chapter 7, 2023) [47]. The proposed method can be applied to any number of chemicals with the known concentration–response relationship and confidence interval for each substance.

According to the methodology presented above, the only way to determine non-additive toxicity is through toxicity testing; however, it is unfeasible to test every possible combination of chemicals in specific environmental conditions. This obstacle is also relevant for real environmental conditions with many contaminants at a low concentration. The solution for such a situation might be found using the funnel hypothesis, which states that non-additive effects (synergistic and antagonistic) can be observed only for mixtures with few chemicals, and additive effects dominate with an increase in the number of chemicals [50]. The funnel hypothesis assumes that in the case of a large number of chemicals, the synergistic and antagonistic effects are nullified, and the overall effect becomes additive [43,47,51]. However, this assumption is controversial and several studies have been suggested that synergistic effects are more likely to appear in multicomponent systems than additive ones [52,53,54].

Apart from classical CA and IA models, the toxicity of environmental contaminants can be predicted by the models based on quantitative structure–activity relationship (QSAR) techniques [43,44]. QSAR is the similarity-based statistical technique that identifies the mathematical relationship between the molecular properties (activity/toxicity/physical property) and structural features of molecules [55]. This approach aims to overcome the limitations of conventional models, such as the lack of knowledge in the MoAs for many chemicals and the inability to predict synergistic effects. Moreover, QSAR-based computational models can find relationships between theoretically derived molecular descriptors and empiric toxicological responses [56]. QSAR models have been used for the toxicity assessment of different nanomaterials, such as metal oxides, silica nanoparticles, and carbon nanotubes [54,55,56]. For instance, research has been conducted on multi-walled carbon nanotubes, fullerenes, and other CNMs to understand their potential toxic effects on living organisms and the environment [54]. Additionally, research by Buglak et al. (2019) discussed the application of QSAR in cytotoxicity studies of various engineered nanomaterials, including multi-walled carbon nanotubes, highlighting the effectiveness of QSAR models in toxicological assessments [56].

The successful development and application of QSAR-based modeling for mixture toxicity assessment will be further stimulated by the recent rise of computation capacity and the growing body of experimental data.

### 3.2. Applicability of Joint Toxicity Models for Carbon-Based Nanomaterials

While previous studies have successfully applied CA, IA, and QSAR-based models to mixtures of organic chemicals, there is a gap in predictive models for the mixtures, including nanomaterials. Currently, few studies have been applied using predictive models to assess the joint toxicity of CNMs with common contaminants, and there is a need for further research in this area.

CA and IA models were used for the prediction of the joint toxicity of graphene, GO, and five ionic liquids in the freshwater green microalga *S. obliquus* [57]. The other work applied a QSAR-based model to predict the combined toxicity of graphene nanoplatelets with 3,4-dichloroaniline on microalgae *S. obliquus* and *C. pyrenoidosa* [58]. In their work, Zhang et al. (2021) presented the integration of classic approaches to mixture toxicity assessment and in silico methods for forecasting the toxicity of a mixture of engineered nanoparticles [59]. The nano-mixture QSAR models, with the application of different machine learning techniques (i.e., random forest, neural network, support vector machine, and multiple linear regression), have been developed by Trinh et al. (2021) to predict the joint toxicity of TiO_2_ nanoparticles and four metal ions in *Daphnia magna* [60]. This approach of nano-mixture QSAR modeling has good potential to be applied for CNMs.

Several works used QSAR models to predict the adsorption of different contaminants by CNMs. Apul et al. (2013) developed predictive models based on QSAR and Linear Solvation Energy Relationship (LSER) techniques for the adsorption of 29 aromatic contaminants by CNTs [61]. Lata and Vikas (2021) in their work proposed a quantum-mechanical model to predict the concentration-dependent adsorption coefficients of aromatic and aliphatic organic contaminants by graphene nanosheets [62].

These studies demonstrate the significance of QSAR models in forecasting the toxic interactions of CNMs with different contaminants, providing valuable insights for risk assessment and the safe design of nanomaterials. Along with the need for higher computational power, further progress in this area is restricted by limited experimental data on the joint toxicity of CNMs with different co-contaminants in aquatic species.

The next section summarizes the existing research data on the interaction and mixture toxicity of CNMs with common environmental pollutants.

## 4. Current Available Data of CNMs Interaction and Joint Toxicity with Emerging Aquatic Pollutants

This section gathers and discusses the main existing studies devoted to the interaction and aquatic toxicity of CNMs with common pollutants, such as heavy metals, pesticides, hydrocarbons, and others.

Literature collection was performed in Google Scholar (scholar.google.com) using the keywords “carbon”, “graphene”, “nanomaterial”, “nanoparticle”, “mixture”, “joint toxicity”, and “aquatic species”. Each publication was carefully checked before collection. The gathered publications were annotated to provide the main results of the joint toxicity study. The last search was conducted in July 2024.

**Figure 3:**
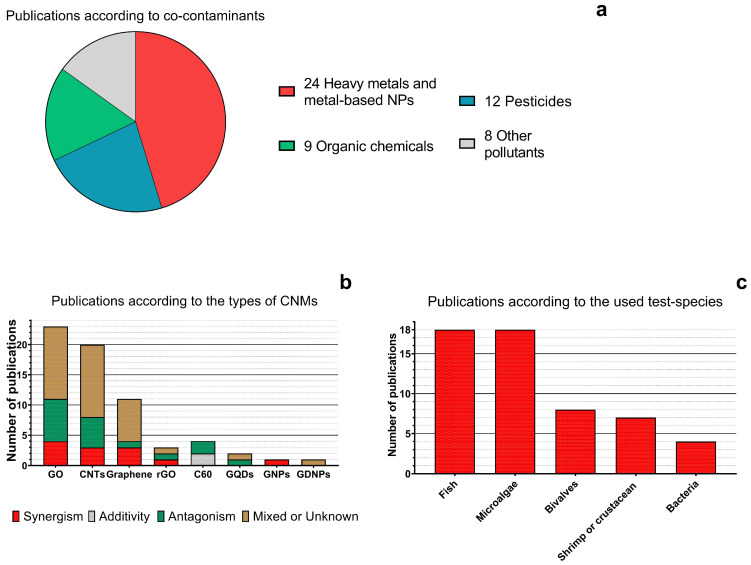
The distribution of the publications devoted to joint toxicity of CNMs: (**a**) based on studied co-contaminants; (**b**) based on used CNMs; (**c**) based on the used test-species. GO, graphene oxide; CNTs, carbon nanotubes; rGO, reduced graphene oxide; C60, fullerene; GQDs; graphene quantum dots; GNPs, graphene nanoplatelets; GDNPs, graphite–diamond nanoparticles.

A total of 53 publications (Figure 3) were sorted and listed in the tables in the following subsections according to used co-contaminants (Figure 3a), such as (1) heavy metals, metal ions, and metal-based nanoparticles (24 studies), (2) pesticides (12 studies), (3) organic chemicals, including hydrocarbons (9 studies), and (4) other emerging pollutants (8 studies). Currently, the most studied CNM (Figure 3b) is GO (23 studies), followed by CNTs (20 studies), and graphene (11 studies). Among the used test models (Figure 3c), the most common were fish and microalgae (18 studies for each). The most used species was zebrafish *Danio rerio* (12 studies).

### 4.1. Joint Toxicity of CNMs with Heavy Metals and Metal-Based Nanoparticles

Heavy metals in very low concentrations are found naturally occurring in the Earth’s crust and often have an essential role in maintaining various biochemical and physiological functions in living organisms [63]. However, human activities have led to a dramatic increase in metal concentrations in water and provoked serious ecotoxicological risks for aquatic species [64,65]. Among the heavy metals, essential elements include copper (Cu), selenium (Se), chromium (Cr), molybdenum (Mo), zinc (Zn), and iron (Fe), but these elements become toxic above certain threshold concentrations. Non-essential heavy metals are cadmium (Cd), lead (Pb), and mercury (Hg), and these elements can replace essential metals and exhibit high toxicity by accumulation at metabolically active sites [63].

The metals released into the aquatic environment are distributed between an aqueous phase and sediment. Suspended trace metals exist as free ions, either as ions bound to different ligands or ions absorbed to the surface of suspended particles and organic matter [66]. Moreover, the interaction with different compounds and other pollutants can alter the toxic effects of heavy metals.

According to their high surface area and porosity, CNMs have been considered as promising adsorbents for heavy metal ion removal [67]. Moreover, CNMs have great potential for functionalization, which allows the adjustment of their surface properties for the better absorption of specific pollutants [68]. The maximum sorption capacity of carbon materials for toxic metal removal was evaluated with the Langmuir isotherm and pseudo-second-order kinetic models, and CNMs were listed in the following order beginning from the highest sorption: carbon nanotubes > graphene > activated carbon > carbon quantum dots [69]. The adsorption processes are influenced by factors like pH, temperature, contact time, and dosage [67,69]. In general, the application of pure CNMs and CNM-based adsorbents for the removal of heavy metals and other pollutants from aqueous systems has demonstrated high efficiency in many laboratory-scale studies [70,71,72], but it requires further testing in the conditions of real-life industrial wastewater, which is complicated by the presence of other pollutants and organic compounds. Moreover, there is a risk of secondary water contamination by CNMs with bounded metal ions which could cause a significant threat to aquatic species.

It should be highlighted that the toxic level of most types of CNMs for aquatic species is relatively lower than that of heavy metal ions and metal-based nanoparticles [73,74]. Moreover, the toxicity varies depending on species sensitivity to the impact of different heavy metals [75,76,77] and different types of CNMs [78,79]. To date, a few studies have investigated the joint toxicity of these pollutants in aquatic environments and the mechanisms of their combined toxic action are not fully understood. Most of the existing studies reported antagonistic interactions between CNMs and metal ions. The existing publications related to the aquatic toxicity and interaction of CNMs with heavy metals, metal oxides, and metal-based nanoparticles are represented in Table 1.

### 4.2. Joint Toxicity of CNMs with Pesticides

Pesticides are broadly applied to increase agriculture production and to fight against disease-causing vectors. Pesticides generally include herbicides, insecticides, algaecides, antimicrobials, repellents, fungicides, and others [103]. Pesticides are chemically classified as organochlorines, organophosphate, carbamates, and substituted ureas. Among these, organochlorines are the most hazardous class of pesticides, followed by organophosphorus and carbamates [104]. At the same time, pesticides or pesticide residues may cause various harmful effects on living organisms and the environment. Through spillage, industrial effluent, surface runoff, or pesticide-treated soils, pesticides or pesticide residues are transported into the aquatic environment where aquatic organisms are extremely vulnerable to their toxicity [105,106].

The comprehensive review work of Hegde et al. (2024) summarized the state of the art in agricultural applications of CNMs, such as agrochemical sensing, agrochemical remediation, and fertilizer delivery [107]. Many research works demonstrated that CNMs can act as carriers, facilitating the entry of chemicals into organisms, which fit a phenomenon known as the ‘Trojan-horse effect’ [108]. This effect might have a positive outcome and is often applied intentionally for an enhancement of pesticide target reaching and lowering possible negative effects for other organisms [109]. At the same time, there are cases with opposite results.

Considering the diversity of pesticides, understanding the combined toxicity of CNMs and pesticides is crucial for assessing and mitigating their effects on the aquatic environment. The existing publications that are related to aquatic toxicity and the interaction of CNMs with pesticides are represented in Table 2.

### 4.3. Joint Toxicity of CNMs with Organic Contaminants, Including Hydrocarbons

Hydrocarbon contamination, which includes compounds like aliphatic, monoaromatic (BTEX), polycyclic aromatic hydrocarbons (PAHs), phenols, and volatile organic compounds, discharges into water bodies from sources like crude oil, petroleum-based products, and pesticides [122]. Petroleum industries contribute significantly to hydrocarbon contamination through oil spillage during exploration, transportation, storage, and refining processes.

Hydrocarbon contamination levels in water vary across different regions. Research on the Danube River and its branches in Hungary revealed concentrations of PAHs ranging from 25 to 1208 ng/L in water samples and 8.3 to 1202.5 ng/g in sediments, with pyrogenic sources identified as major contributors [123]. The total average concentrations of PAHs in seawater, surface sediment, and marine organisms of Haizhou Bay, China, were 24.8 ng/L, 293.5 ng/g, and 392.6 ng/g, respectively [124]. Studies in Nigeria have shown elevated total petroleum hydrocarbon (TPH) concentrations in water, sediment, and fish samples, with the highest levels observed in sediment samples [125]. These findings underscore the urgent need for remediation efforts to mitigate hydrocarbon pollution in water bodies and protect both the environment and human health.

Different types of CNTs were reported in removing benzene, toluene, ethylbenzene, and xylene (BTEX) from an aqueous solution with better adsorption capacity for SWCNTs [126]. Purified CNTs with opened ends have more adsorption capacity [127]. The other effective method to enhance the performance of CNTs for BTEX adsorption is surface modification [128]. Graphene and GO nanosheets were described as effective adsorbents for PAH removal [129]. GO nanoparticles demonstrated higher adsorption affinity to PAHs compared to CNTs and C60 nanoparticles due to reduced aggregation and the high surface O-content of GO nanoparticles [130].

The existing publications related to aquatic toxicity and the interaction of CNMs with hydrocarbons and other organic contaminants are represented in Table 3.

### 4.4. Joint Toxicity of CNMs with Other Co-Contaminants

This subsection summarizes the studies of CNM interaction with other contaminants that have not been represented above. It includes chemical derivatives, antibiotics, and binary interaction between different CNMs. Table 4 lists the publications related to toxicity and the interaction of CNMs with aquatic contaminants not represented in previous tables.

## 5. Conclusions and Future Prospects

The impressive rise of nanotechnology, including the growth in the production and application of CNMs, supports the development in optics, energy storage, healthcare, and other areas of industry and daily life. At the same time, the widespread application of CNMs includes risks to human health and the environment. Despite the safety of the most common CNMs, such as CNTs, graphene, or GO, which have been examined in different aquatic and terrestrial species for two decades, the mechanisms of their toxic action are not fully understood. Moreover, the risk assessment of nanomaterials is further complicated by the diversity of their types and surface modifications, environmental transformation, and interaction with other chemicals.

The existing body of research data reveals the lack of standardized assays, which makes the comparison of studies on CNM mixture toxicity difficult. This review set out to provide and discuss the state of the art in principles of combined toxicity assessment and the joint toxic action of CNMs with other pollutants in aquatic species.

The developed surface area and the possibility of surface functionalization allow CNMs to effectively adsorb metal ions, pesticides, and other organic compounds. Alongside the possible application for water purification, it changes the bioavailability of the pollutants to aquatic species. Whether joint action will be antagonistic or synergetic to a large extent depends on the hydrophilic/hydrophobic properties of the CNMs and cell membrane properties of the exposed organism. For example, it was demonstrated that oxidized CNTs had additive toxicity with hydrophobic organochlorine pesticide pentachlorophenol and a synergistic toxic effect with hydrophilic antibiotic ciprofloxacin in the bacteria *B. subtilis* [121].

Moreover, CNMs can increase the uptake of adsorbed pollutants by the phenomenon called the “Trojan horse effect”. However, further toxic action depends on the ability of the CNM to release the adsorbed chemical. It was demonstrated that oxidized MWCNTs increased the uptake and toxicity of Cd in the *D. rerio* liver cell line [84], but GO reduced the toxicity of Cu^2+^ to the microalgae *S. obliquus* [90] and *C. pyrenoidosa* [93] and the bacteria *E. coli* and *S. aureus* [98]. The other observed trend is the synergistic action at low concentrations caused by the “Trojan horse effect” and the antagonism at high doses of CNMs caused by an aggregation of carbon nanoparticles which had already adsorbed a pollutant [118]. However, the effect might be the opposite if the CNM has higher toxicity than the other chemical or material in the co-exposure [85].

This review has also shown that the great variability of the parameters that dramatically influence the real-life behavior and toxicity of CNMs cannot be considered in any experimental study. Thus, a risk assessment bioassay should focus on the attempt to find patterns in the interaction of different isolated parameters of the assay. From this perspective, further research studies with different aquatic species exposed to CNMs combined with various aquatic pollutants are needed to gather more evidence for further analytical processing.

The other suggestion for future study includes the development, adaptation, and improvement of in silico computation models for the joint toxic action of CNMs in aquatic media. Despite several successful applications of QSAR-based models for studying the combined toxicity [57] and adsorption [61,62] of CNMs with other compounds, this research area is still in the very early stages of development. These models will allow analysis of the existing in vivo risk assessment data and will help to develop the forecasting ability for untested combinations of pollutants.

## Figures and Tables

**Table 1:** Summary of current studies on CNMs co-exposure with heavy metals, metal oxides, and metal-based nanoparticles in aquatic species.

№	CNMs	Co-Contaminant	Species	Toxicity Endpoints	Observed Effects	Reference
*Studies used fish as a test-model*						
1.	GN	Two types of nanocomposites with Ni	*Danio rerio*	3 h, 144 h post-fertilization embryo toxicity test, biochemical response, locomotor behavior assay, bioaccumulation	neither of two GN/Ni nanocomposites presented lethal or developmental effects in zebrafish; both nanocomposites reduced the locomotion of zebrafish larvae; the differences in biochemical response were mostly associated with shape of nanoparticles than with their size	Almeida et al., 2019 [80]
2.	Multilayer GN	ZnO	*Capoeta fusca*	96 h LC50, histopathological and behavioral effects	synergistic at 96 h acute exposure, antagonistic effect on the histopathological and behavioral disorders	Sayadi et al., 2022 [81]
3.	GO	Zn, Cd	*Geophagus iporangensis*	24 h metabolic rate, ammonia excretion	GO intensified metabolic rise and ammonia excretion in fish caused by Zn, co-exposure of GO and Cd only decreased metabolic rate and did not affect ammonia excretion	Medeiros et al., 2020 [82]
4.	GO	Cr^6+^	*Danio rerio* embryos	48 h exposure, embryo-larval toxicity, bioaccumulation, ROS generation, metabolic changes	co-exposure increased lipid peroxidation in embryos compare to single exposure; GO adsorbed Cr^6+^ ions and enhanced contact between adsorbed Cr^6+^ and chorions; sharp edges of GO also facilitated Cr^6+^ uptake by embryos	Chen et al., 2022 [83]
5.	GO	Mixture of Cr, Cu, Ni, and Zn	*Salmo trutta* (embryos and larvae)	Bioaccumulation, survival, heart rate, genotoxicity, cytotoxicity, metallothionein levels, lipid peroxidation	single and joint exposure had no impact on embryos survival, but lethality of the metal mixture on larvae was nullified in co-exposure with GO; the chorion of embryos was more attracted to GO than external tissues of larvae	Jurgelėnė et al., 2022 [84]
6.	MWCNTs	ZnO NPs	*Cyprinus carpio*	4 weeks of exposure, histopathology, bioaccumulation	antagonistic effect at the low level of MWCNTs and synergetic effect at the high level of MWCNTs; MWCNTs significantly decreased ZnO accumulation in the intestine after four weeks of exposure	Gao et al., 2024 [85]
7.	O-MWCNTs	Cd	*Danio rerio* liver cell line	24 h exposure, DNA comet assay, ROS generation, enzyme activity	synergistic effect; co-exposure increased the Cd content in the cells; two different exposure protocols tested, FBS serum in the culture medium changed the uptake of metal into cells	Morozesk et al., 2020 [86]
*Studies used mussels or clams as test-model*						
8.	GO	Cu	*Ruditapes philippinarum*	29-day exposure, metabolism, and oxidative stress-related parameters	demonstrated the dependence of the toxic response on pH; low pH showed increased electron transport system and glutathione-S-transferase activities and reduced glutathione levels under pollutants co-exposure	Britto et al., 2020 [87]
*Studies used shrimp or crustacean as a test-model*						
9.	GO	Zn, Cd	*Palaemon pandaliformis*	96 h LC50, routine metabolism (oxygen consumption and ammonia excretion)	GO increased the toxicity of Zn and Cd and impaired the routine metabolism of *P. pandaliformis*	Batista de Melo et al., 2019 [88]
10.	GO	Cd^2+^ and BSA (for albumin corona formation)	*Daphnia magna*	48 h EC50 (immobilization)	antagonistic effect; bare GO reduced cadmium toxicity by 110%, albumin coronated GO reduced cadmium toxicity by 238%, albumin corona formation dramatically increased colloidal stability of GO and adsorption capacity of Cd^2+^	Martinez et al., 2020 [89]
11.	SWCNTs, MWCNTs, OH-MWCNTs, COOH-MWCNTs	Cd	*Daphnia magna*	24 h LC50 (immobilization), bioaccumulation	all used CNTs enhanced the toxicity of Cd; the toxicity-increasing effect of SWCNTs and MWCNTs was mainly caused by catalyst impurities, while OH-MWCNTs and COOH-MWCNTs enhanced joint toxicity due to the greater adsorption of Cd	Wang et al., 2016 [90]
*Studies used microalgae as a test-model*						
12.	GN, GO, GN-H	Cd	*Scenedesmus obliquus*	72 h EC50 (growth rate), Chl-a synthesis, cytotoxicity	GN and GO enhanced the toxicity of Cd at all the used concentrations, while GN-H enhanced the toxicity of Cd only at the lowest used concentration (0.1 mg/L); the influence of graphene family NMs on the acute toxicity of Cd was in the order of GO > GN > GN-H (at GNMs concentration 0.1 mg/L to 1 mg/L)	Zhang et al., 2020 [91]
13.	GO	Cu^2+^	*Scenedesmus obliquus*	96 h EC50 (growth rate inhibition) 12 d subacute toxicity test	antagonistic effects; GO reduced the toxicity of Cu even at low and environmentally relevant concentrations (1 mg/L)	Hu et al., 2016 [92]
14.	GO	Cu^2+^	*Chlorella pyrenoidosa*	72 h EC50 (growth rate inhibition), ROS generation	antagonistic effect; pristine GO and Cu^2+^ ions had significantly higher toxic effect than the same chemicals after 8 days of sunlight irradiation; Cu^2+^ ions suppressed the photo-transformation of GO, Cu^2+^ ions formed Cu-based nanoparticles on the photo-transformed GO	Zhao et al., 2020 [93]
15.	rGO	nanocomposites with Au, Ag, Pd, Fe_3_O_4_, Co_3_O_4_, SnO_2_	*Chlamydomonas reinhardtii*, *Scenedesmus obliquus*	96 h acute exposure, ROS quenching, proteomic analysis, membrane damage	microalgae with more hydrophobic cell surfaces had more metal ion adsorption, rGO nanocomposites with more heterointerfaces were more prone to induce cellular oxidative stress and membrane damage	Yin et al., 2020 [94]
16.	GNPs, rGO	nZrO_2_	*Chlorella pyrenoidosa*	72h EC10, EC50 (growth rate inhibition) ROS generation, cellular membrane functional changes	synergistic effect; rGO increased the cytotoxicity and intracellular ROS accumulation to a higher extent than GNPs	Wang et al., 2021 [95]
17.	GQDs	ZnO	*Heterosigma akashiwo*	96 h EC50 (growth rate)	antagonistic effect at low concentrations, and synergistic effect at high concentrations; adsorption of released Zn^2+^ ions on GQDs	Wang et al., 2022 [96]
18.	GQDs	ZnO	*Gymnodinium* sp.	96 h EC50 (growth rate inhibition), ROS generation	antagonistic effect, due to aggregation and sedimentation interaction between nanoparticles; ZnO alone had no negative effect on the algae growth, while GQDs revealed dose-dependent growth rate inhibition	Zhu et al., 2022 [97]
19.	CNTs	CuO	*Skeletonema costatum*	96 h exposure, chlorophyll and photosynthetic efficiency (ΦPSII)	antagonistic effect caused by adsorption of Cu^2+^ on CNTs and aggregation between nano-Cu and CNTs	Zhang et al., 2018 [73]
20.	CNTs	Cu, Cd, Zn	*Scenedesmus obliquus*	96 h EC10, EC50 (growth rate inhibition); 8 d exposure, biochemical response, photosynthetic activity	antagonistic effect caused by inhibition of metal uptake by co-exposure with CNTs; CNTs in single exposure enhanced the photosynthetic activity of *S. obliquus*	Sun et al., 2020 [98]
21.	MWCNTs	CuO	*Scenedesmus obliquus*	96 h EC50 (growth rate inhibition) ROS generation, cell membrane damage	MWCNTs were significantly more toxic than CuO NPs; at lower concentrations, CuO reduced cell membrane damage and ROS level caused by MWCNTs; highest concentrations of MWCNTs and CuO synergistically enhanced the ROS level	Fang et al., 2022 [99]
*Studies used bacteria as test-model*						
22.	GO	Cd^2+^, Co^2+^, Zn^2+^	*Escherichia coli*, *Staphylococcus aureus*	24 h acute exposure	an antagonistic effect caused by metal ions adsorption on GO, an increase in the zeta potential and the size of GO aggregates, and a decrease in the sharpness of GO edge	Gao et al., 2018 [100]
23.	MWCNTs, COOH-MWCNTs, OH-MWCNTs, NH_2_-MWCNTs, SWCNTs	Cu, Cr	microbial communities with dominant *Bacillus* sp. and *Acidithiobacillus* sp.	40 d exposure, population quantitation, microbial community structure, metal ions sorption	co-exposure with metals decreased bacteria population after 10 d exposure, while after 40 d CNTs with Cu, increased bacterial cell number;carboxyl- and hydroxyl-CNTs exhibited more toxicity than pristine SWCNTs, MWCNTs, and amino-functionalized MWCNTs	Wang et al., 2015 [101]
*Studies used multispecies test-model*						
24.	GO	ZnO	*Scenedesmus obliquus*, *Daphnia magna*, *Danio rerio*	EC/LC10, EC/LC50 (algae: 96 h growth rate; daphnids: 48 h immobilization; fish: 96 h lethality)	the joint effects of ZnO NPs and GO NPs were additive to *S. obliquus* and *D. magna* but antagonistic to *D. rerio*. The impact of Zn^2+^-ions was limited due to the adsorption to the GO NPs	Ye et al., 2018 [102]

GN, graphene; GO, graphene oxide; GN-H, amine-modified graphene; GNPs, graphene nanoplatelets; GQDs, graphene quantum dots; rGO, reduced graphene oxide; SWCNTs, single-walled carbon nanotubes; MWCNTs, multi-walled carbon nanotubes; O-MWCNTs, oxidized multi-walled carbon nanotubes; COOH-MWCNTs, carboxylated multi-walled carbon nanotubes; OH-MWCNTs, hydroxylated multi-walled carbon nanotubes; NH_2_-MWCNTs, amino-functionalized multi-walled carbon nanotubes; nZrO_2_, nano-zirconium oxide; BSA, bovine serum albumin.

**Table 2:** Summary of current studies on CNM co-exposure with pesticides in aquatic species.

№	CNMs	Co-Contaminant	Species	Toxicity Endpoints	Observed Effects	Reference
*Studies used fish as a test-model*						
1.	GO	TDCIPP	*Danio rerio*	3 d, 7 d developmental toxicity, mitochondrial function, proteomic assays	antagonistic effect on the developmental toxicity (malformation, mortality, and heart rate), GO co-exposure promoted activation of the energy metabolisms in zebrafish and mitigated the adverse effects induced by TDCIPP	Zou et al., 2020 [110]
2.	MWCNTs, COOH-MWCNTs	bifenthrin	*Danio rerio*	42 d experiment (28 d exposure phase and 14 d elimination phase), gene expression, bioaccumulation	MWCNTs and COOH-MWCNTs increased the impact of bifenthrin on zebrafish; the genes related to immunity, endocrine activity, and neurotoxicity showed enantioselective expression in different zebrafish tissues; sex-specific differences were observed	Zhao et al., 2022 [111]
3.	MWCNTs	BDE-47	*Danio rerio*	2 h embryo, 96 h LC50; embryonic development, oxidative stress, apoptosis, DNA damage	antagonistic effect, BDE-47 induced development inhibition, oxidative stress, and apoptosis in zebrafish; MWCNTs limited bioavailability of BDE-47, the levels of oxidative stress biomarkers, apoptosis, and DNA damage decreased in the presence of MWCNTs	Wang et al., 2020 [112]
4.	HNO_3_–MWCNT	carbofuran	*Oreochromis niloticus*	96 h LC50; oxygen consumption, swimming behavior	synergistic effect, HNO_3_–MWCNT more than five-fold increased the acute toxicity of carbofuran; co-exposure caused a decrease in both oxygen consumption and swimming capacity	Campos-Garcia et al., 2015 [113]
*Studies used mussels or clams as test-model*						
5.	GN	TPP	*Mytilus galloprovincialis*	computational toxicology and multi-omics technology	the down-regulated genes in graphene + TPP treatment were mainly associated with oxidative stress and energy metabolism; metabolic response indicated disturbances in energy metabolism and osmotic regulation under co-exposure	Li et al., 2021 [114]
6.	GN	TPP	*Mytilus galloprovincialis*	embryo exposure, in silico toxicogenomic, metabolic pathway analysis, oxidative stress, developmental abnormality	authors established a conceptual framework of developmental abnormality; co-exposure induced significant transcriptional inhibition, disturbed morphology and physiological parameters, increased deformity and mortality to induce the developmental abnormality	Wang et al., 2023 [115]
7.	GN	TPP	*Mytilus galloprovincialis* hemocytes	hematotoxicity, genotoxicity, oxidative stress	GN exposure caused oxidative stress and DNA damage in the hemocytes and these effects were significantly reduced after combined exposure with TPP; the up-regulated genes in the co-exposure group were mainly associated with reduced apoptosis and DNA damage	Meng et al., 2020 [116]
*Studies used shrimp or crustacean as a test-model*						
8.	GO	PYR, LCT	*Daphnia similis*	48 h EC10, EC50 (immobilization), uptake	synergistic effect, Trojan horse effect; GO increased toxicity up to 83% for PYR and 47% for LCT, pesticide adsorption on GO led to the stabilization of the suspensions; properties of the organic toxicants can influence the stability of graphene oxide suspensions and plays a fundamental role in the modulation of their toxicity	de Paula et al., 2022 [117]
9.	GDNPs	TBZ	*Daphnia magna*	EC50 (48 h, immobilization)	synergism at low concentrations (probably the ‘Trojan horse’ effect) and antagonism at high GDNPs doses caused by aggregation of GDNPs and reducing the bioavailability of adsorbed TBZ	Martín-de-Lucía et al., 2019 [118]
10.	HNO_3_-MWCNT	carbofuran	*Palaemon pandaliformis*	24 h exposure, metabolic rate (oxygen consumption), and ammonia excretion	higher increase in metabolic rate and ammonia excretion after co-exposure (probably additive effect)	Alves et al., 2022 [119]
*Studies used bacteria as test-model*						
11.	CNTs	PCP	*Escherichia coli*	Bacterial growth inhibition, cell morphology changes, oxidative stress, transcriptional changes, bioaccumulation	antagonistic toxicity; PCP decreased CNT bioaccumulation; CNTs attenuated the PCP-induced disturbances of gene expression in biosynthetic, protein metabolic, and small molecule metabolic processes	Deng et al., 2019 [120]
12.	O-CNTs	PCP, CIP	*Bacillus subtilis*	3 h EC50 (bacterial growth), ROS generation, metabolomic response	additive effect with hydrophobic PCP and synergistic effect with hydrophilic antibiotic CIP because of ‘Trojan horse effect’; CNTs, PCP, and CIP had similar influences on the contents of fatty acids, amino acids, glycerol, galactosamine, and small molecular acids in bacteria	Deng et al., 2021 [121]

GN, graphene; GO, graphene oxide; GDNPs, graphite–diamond nanoparticles; HNO_3_-MWCNT, multi-walled carbon nanotubes oxidized with HNO_3_; MWCNTs, multi-walled carbon nanotubes; COOH-MWCNTs, carboxylated multi-walled carbon nanotubes; O-CNTs, oxidized carbon nanotubes; CIP, ciprofloxacin; LCT, lambda-cyhalothrin; PCP, pentachlorophenol; PYR, pyriproxyfen; TBZ, thiabendazole; TDCIPP, tris(1,3–dichloro–2–propyl) phosphate; BDE-47, 2,2′,4,4′-tetrabromodiphenyl ether; TPP, triphenyl phosphate.

**Table 3:** Summary of current studies on CNMs co-exposure with hydrocarbons and other organic contaminants.

**№**	**CNMs**	**Co-Contaminant**	**Species**	**Toxicity Endpoints**	**Observed Effects**	**Reference**
*Studies used fish as a test-model*						
1.	GO	BPA	*Danio rerio* embryo, larvae, and adult male fish	7 d exposure, deep neural network modeling, molecular docking analysis, metabolic pathway analysis	GO enhanced the endocrine disruption effects of BPA in the adult zebrafish by the significant reduction in testosterone and follicle-stimulating hormone levels, and lowering spermatozoa; co-exposure caused disturbance in three additional metabolic pathways and stronger perturbations on carbohydrate, lipid, and amino acid metabolism in adult fish; the opposite effect observed in zebrafish embryo and larvae	Chen et al., 2022 [131]
2.	SWCNT	PFOS	*Danio rerio*	24, 48, 72, and 96 h exposure, bioaccumulation, AChE activity, ROS generation, antioxidation enzymes	enhanced the injury effect of PFOS on ROS, SOD, CAT, and AChE activity; PFOS was adsorbed by SWCNT, which reduced the bioconcentration in zebrafish tissue and enhanced that in skin	Li et al., 2017 [132]
3.	SWCNTs, MWCNTs	a mixture of different-type CNTs, NOM	*Danio rerio*	96 h survival, embryo development, oxidative stress, transcriptional effects	embryonic chorions had a stronger barrier to the mixed-type CNTs than to the single-type CNTs, but the presence of NOM weakened this barrier; NOM reduced the antioxidant activity and the expression of genes involved in the antioxidant pathway	Lu and Wang 2023 [133]
4.	MWCNTs	fluoranthene and NOM	*Pimephales promelas*	16 h exposure, bioavailability, bioaccumulation	bioavailability of fluoranthene was reduced after adsorption to MWNTs, from 60% to 90% of the fluoranthene was adsorbed to the MWNTs; fluoranthene was not desorbed from ingested MWCNTs; NOM influenced the adsorption of fluoranthene to MWNTs	Linard et al., 2014 [134]
*Studies used mussels or clams as test-model*						
5.	GN	TPP	*Mytilus galloprovincialis*	7 d exposure, gene expression, enzyme activity	TPP adsorption on GN could inhibit the surface activity of GN and reduce tissue damage and oxidative stress; GN in single up-regulated exposure the expression of the stress response, cytoskeleton, and reproductive genes, but these genes were significantly down-regulated after combined exposure	Meng et al., 2019 [135]
6.	GO	B[a]P	*Mytilus galloprovincialis*	7 d exposure, bioaccumulation, hemocyte response, enzyme activities in tissues, histopathology	higher joint toxicity due to the “Trojan horse” effect, but bioaccumulation of BaP was reduced by GO nanoplatelets	González-Soto et al., 2023 [136]
7.	C60	B[a]P	*Mytilus galloprovincialis*	72 h exposure, genotoxic and proteomic response	the antagonistic effect at the genotoxic and proteomic level was observed based on a single concentration of C60 (further study is needed); co-exposure caused no difference in bioaccumulation and no Trojan horse effects	Barranger et al., 2019 [137]
8.	C60	fluoranthene	*Mytilus* sp.	72 h exposure, oxidative stress, genotoxicity, histopathology, physiological effects	co-exposure had rather additive than synergistic effects; co-exposure enhanced the levels of DNA strand breaks and elevated total glutathione levels indicating oxidative stress	Al-Subiai et al., 2012 [138]
*Studies used microalgae as a test-model*						
9.	GN, GO, rGO	HA	*Chlorella pyrenoidosa*	ROS generation	antagonism between HA and all the three types of NMs; the degree of antagonism followed the order rGO > GO > GN; HA reduced membrane damage and in microalgae and NMs–algae heteroaggregation (for rGO and G)	Zhao et al., 2019 [139]

GN, graphene; GO, graphene oxide; rGO, reduced graphene oxide; C60, fullerene; CNTs, carbon nanotubes; BPA, bisphenol A; PFOS, perfluorooctane sulfonate; NOM, natural organic matter; TPP, triphenyl phosphate; B[a]P, benzo(a)pyrene; HA, humic acid; AChE, acetylcholinesterase.

**Table 4:** Summary of current studies on CNMs co-exposure with the other emerging aquatic contaminants.

№	CNMs	Co-Contaminant	Species	Toxicity Endpoints	Observed Effects	Reference
*Studies used fish as a test-model*						
1.	C60, SWCNTs, MWCNTs, GO, GN	As (III)	*Danio rerio*	96 h acute exposure, As accumulation, biochemical responses	GO and GN elevated accumulation and toxicity of As (III) in *D. rerio*, while the effect was marginal for co-exposure to SWCNTs, MWCNTs, and C60	Wang et al., 2021 [140]
2.	C60, SWCNTs, MWCNTs, GO, GN	As (V)	*Danio rerio*	96 h acute exposure, As accumulation, biochemical responses	C60 reduced the toxicity of As(V) probably due to coating As(V) ion channels and inhibition of total As accumulation; MWCNTs demonstrated a similar C60 effect, while accumulation and toxicity of As(V) had little or no change in the presence of SWCNTs, GO and GN	Wang et al., 2024 [141]
*Studies used microalgae and cyanobacteria as test-model*						
3.	GN, GO	Five ionic liquids	*Scenedesmus obliquus*	EC10, EC50 (96 h growth rate inhibition)	additive effect at low concentrations of the mixtures but antagonistic at high concentrations; a combination of GO with ionic liquids had more severe joint toxicity than the binary mixtures with GN; the mechanism of the joint toxicity may be associated with the adsorption capability of the graphenes for the ionic liquids	Wang et al., 2017 [57]
4.	GO	As (III), As (V)	*Chlorella pyrenoidosa*	72 h EC50 (growth rate inhibition), ROS generation, membrane damage	a synergistic toxic effect between GO and As (III, V); even at environmental concentrations of As (III, V), the adsorption capacity of GO for As (III) was higher than As (V)	Cao et al., 2019 [142]
5.	GO	biologically treated wastewater	*Chlamydomonas reinhardtii*	72 h-EC50 (growth rate), esterase activity, cytoplasmic membrane potential, ROS generation	the antagonistic effect; joint exposure significantly reduced cytotoxicity due to the adsorption of toxic chemicals on the surface of GO nanoparticles and to the higher aggregation of GO in wastewater	Martín-de-Lucía et al., 2018 [143]
6.	GO	FLO, ETM, OFL, CTC	*Synechocystis* sp.	96 h exposure, ROS quenching, membrane permeability, malondialdehyde analysis, proteomic analysis	additive effect with FLO, antagonistic effect with ETM, OFL, and CTC; combined exposure groups revealed increased membrane permeability due to down-regulation of the proteins related to perceiving and transmitting the signals of hyperosmotic stress	You et al., 2022 [144]
7.	GO	GOQD, C-SWCNT	*Microcystis aeruginosa*	72 h, 7 d growth inhibition, ROS generation, metabolomic response	antagonistic action of the GO+C-SWCNT mixtures and synergistic action for the GO+GOQD mixture; a hormetic effect on microalgae proliferation was observed for GOQD and the GO+GOQD mixture	Zhao et al., 2023 [145]
8.	CNTs	CAP, TC	*Synechocystis* sp.	96 h acute exposure, ROS generation	additive effect in CNTs+CAP co-exposure; CNTs mitigated the inhibition effect of CAP on protein biosynthesis, while CAP enhanced the up-regulation of proteins induced by CNTs; antagonistic effect in CNTs+TC exposure due to the strong adsorption and catalytic degradation of TC by CNTs	You et al., 2021 [146]

GN, graphene; GO, graphene oxide; C60, fullerene; SWCNTs, single-walled carbon nanotubes; MWCNTs, multi-walled carbon nanotubes; CNTs, carbon nanotubes; C-SWCNT, carboxylic acid-functionalized single-walled carbon nanotubes; GOQD, graphene oxide quantum dots; As (III), arsenite; As (V), arsenate; FLO, florfenicol; ETM, erythromycin, OFL, ofloxacin; CTC, chlorotetracycline; CAP, chloramphenicol; TC, tetracycline.

## Data Availability

Not applicable.
